# Tramadol/Acetaminophen Combination Tablets in Cancer Patients with Chemotherapy-Induced Peripheral Neuropathy: A Single-Arm Phase II Study

**DOI:** 10.1089/pmr.2020.0031

**Published:** 2020-04-30

**Authors:** Daisuke Naruge, Fumio Nagashima, Kirio Kawai, Naohiro Okano, Takaaki Kobayashi, Junji Furuse

**Affiliations:** Department of Medical Oncology, Kyorin University Faculty of Medicine, Tokyo, Japan.

**Keywords:** acetaminophen, anticancer chemotherapy, chemotherapy-induced peripheral neuropathy, CYP2D6, tramadol

## Abstract

***Background:*** Chemotherapy-induced peripheral neuropathy (CIPN) is a frequent complication in patients receiving anticancer chemotherapy, but no effective treatment is yet available.

***Objective:*** To evaluate the efficacy and safety of a tramadol/acetaminophen combination tablets for CIPN.

***Design:*** This is a single-arm phase II study of tramadol/acetaminophen.

***Setting/subjects:*** Eligible patients had received oxaliplatin, paclitaxel, or nab-paclitaxel, and were experiencing CIPN. The patients were given one tablet (37.5 mg tramadol plus 325 mg acetaminophen) twice a day for 7 days, then four times a day for 21 days.

***Measurements:*** The primary endpoint was the numerical rating scale of neuropathic pain. Other endpoints included the potential of CYP2D6 genetic variants to effective response or toxicity.

***Results:*** Of the 34 patients enrolled, 23 completed the protocol treatment. The mean neuropathic pain score decreased insignificantly from 5.53 at baseline to 5.00 at 28 days (95% confidence interval −0.21 to 1.43; *p* = 0.139). However, 13 of the 23 (56.5%) patients who completed the protocol treatment showed improvement of the neuropathic pain score by at least 1 point. No severe adverse events were observed. Tramadol/acetaminophen may be more effective in patients with the intermediate metabolizer phenotype of the CYP2D6 single nucleotide polymorphisms (SNPs) although at the cost of increased toxicity.

***Conclusions:*** Although tramadol/acetaminophen tablets did not reduce neuropathic pain to a statistically significant degree, the neuropathic pain severity reduced in more than a half of the patients.

## Introduction

Supportive care for chemotherapy-related adverse effects is vitally important to improving the survival and quality of life of cancer patients. Several anticancer drugs frequently cause chemotherapy-induced peripheral neuropathy (CIPN).^1^ Oxaliplatin (L-OHP),^2^ paclitaxel (PTX), and nanoparticle albumin-bound paclitaxel (nab-PTX)^3^ are important agents in the treatment of unresectable gastrointestinal cancers but are severely neurotoxic. Their administration is usually continued for more than six months, and in many cases, the symptom of CIPN lasts after graduating anticancer chemotherapy. CIPN not only impacts upon patient quality of life, but also affects the efficacy of cancer chemotherapy, as dose-limiting toxicity may necessitate chemotherapy reduction or discontinuation. Although many agents have been examined for their efficacy against CIPN in clinical trials, only the serotonin–noradrenaline reuptake inhibitor (SNRI) duloxetine has to date been demonstrated to show some efficacy against CIPN in a phase III study.^4^ The efficacy of duloxetine is not clinically robust, however, and the development of more effective treatments for CIPN is becoming more urgent.

Tramadol hydrochloride not only serves as a μ-opioid receptor agonist, but also acts as an SNRI, activating the descending pain-inhibitory pathways.^5^ Acetaminophen is metabolized to AM404, which also activates the descending pain-inhibitory pathways through the cannabinoid receptors.^6^ Tramadol/acetaminophen combination is, therefore, held to exert a synergistic effect against neuropathic pain, and is generally used for the treatment of neuralgias (e.g., spinal canal stenosis and diabetic neuropathy).^7^ Although the tramadol/acetaminophen combination tablet (TACT) may be expected to reduce the severity of the symptoms in CIPN, only one clinical trial of TACT against CIPN limiting to L-OHP has been reported to date.^8^

Cytochrome P450 2D6 (CYP2D6) metabolizes tramadol to *O*-desmethyltramadol (M1). Many SNPs of *CYP2D6* have been reported.^9,10^ Although *CYP2D6* genetic variants have the potential to become useful biomarkers of the efficacy and/or toxicity of tramadol, no clinical study for CIPN have been conducted. And CYP2D6 does not affect the metabolism of acetaminophen.

Hypothesizing that the TACT would be effective for reducing the neuropathic pain of CIPN, we conducted this phase II study to evaluate its efficacy and safety against CIPN. We also investigated genetic variations in *CYP2D6* as a predictive marker of the efficacy and toxicity of TACT.

## Patients and Methods

### Patients

This study was conducted in a single center, Kyorin University Hospital. Eligible patients were those who had previously received or were currently receiving anticancer chemotherapy, which included L-OHP, PTX, or nab-PTX, and had at least grade 2 peripheral sensory neuropathy, diagnosed based on the National Cancer Institute Common Terminology Criteria for Adverse Events version 4.0 (CTCAE v4.0), persisting for more than or equal totwo weeks. Other eligibility criteria included age ≥20 years, an Eastern Cooperative Oncology Group performance status of 0 to 2, and capability of oral intake for at least 4 weeks. Concomitant use of nonsteroidal anti-inflammatory drugs (NSAIDs), other opioids, or adjuvant analgesics (e.g., pregabalin, goshajinkigan, antidepressants, anticonvulsants, and antiarrhythmic agents) for cancer-related pain was allowed if the pain control was stable and the dosages were not expected to be changed during the study period. Exclusion criteria were peripheral neuropathies induced by other causes (e.g., spinal stenosis, diabetes mellitus, alcohol, and other drugs), unstable control of cancer-related pain, uncontrolled nausea/vomiting, and pregnancy/lactation. The study design complied with the Helsinki Declaration and all patients in this study provided written informed consent. This study was conducted with the approval of our institutional ethics committee and was registered with the University Hospital Medical Information Network Clinical Trials Registry (protocol ID UMIN000018147).

### Treatment plan

This trial was a single-arm phase II study. Each TACT contained tramadol hydrochloride 37.5 mg plus acetaminophen 325 mg. The total duration of protocol treatment with TACT was 28 days. The participants took one tablet twice daily, after breakfast and before sleep, for the first 7 days, with the dose increased to four tablets a day, taken after each meal and before sleep, for the subsequent 21 days. Continuation of TACT after the protocol treatment period of 28 days was permitted if the patient felt that it was effective against CIPN and was willing to continue the treatment. As prophylaxis against nausea and vomiting, which is reported as the most common side effect of tramadol/acetaminophen,^7^ prochlorperazine maleate 5 mg was administered three times a day, after each meal, for the first 14 days.

### Endpoints

Primary endpoint was the change in a numerical rating scale of neuropathic pain (NRSN) according to CIPN from baseline (day 0) to day 28 after the start of protocol treatment. The NRSN was reported by patients on the basis of the average severity of the neuropathic pain during the previous three days: 0 point representing no neuropathic pain and 10 point representing neuropathic pain of the worst imaginable severity.

Secondary endpoints were the clinical grade of peripheral sensory neuropathy and the safety of TACT during the treatment period. The clinical grade of peripheral sensory neuropathy and adverse events (AEs) of TACT were recorded in accordance with CTCAE v4.0. The peripheral sensory neuropathy was graded from 0 to 5 (grade 0, no symptoms; grade 1, asymptomatic with loss of deep tendon reflexes or paresthesia; grade 2, moderate symptom severity limiting instrumental activities of daily living (ADL); grade 3, severe symptom severity limiting self-care ADL; grade 4, life-threatening consequences; grade 5, death) in the worst severity during the period from last visit.

NRSN and clinical grade of the peripheral sensory neuropathy were determined at baseline and on days 7, 14, and 28. Any AEs were recorded at each scheduled visit and on any unscheduled visits.

### Examination of CYP2D6 SNPs

As exploratory endpoints, the SNPs of *CYP2D6* were assessed to evaluate their correlations with the efficacy and toxicity of TACT. Participants who provided consent to undergo this biomarker research were included in the analysis of the SNPs. The SNPs were measured using DNA extracted from white blood cells in a 3-mL sample of whole blood. Exploration for *CYP2D6* variations was performed using xTAG CYP2D6 Kit v3 RUO (Luminex Molecular Diagnostics, Canada), which is valid for checking the SNPs of *CYP2D6* allele name *1 to *12, *14, *15, *17, *29, *35, *41, and duplication. The many genetic variants of *CYP2D6* have been classified into the following four phenotypes by the combination of each allele: poor metabolizers (PMs), intermediate metabolizers (IMs), extensive metabolizers (EMs), and ultrarapid metabolizers (UMs).

### Statistical analysis

The primary endpoint was defined as the mean change in NRSN from baseline to day 28. In the previous study of duloxetine,^5^ the mean neuropathic pain score (standard deviation [SD]) at baseline in the duloxetine and control groups was 5.6 (1.6) and 6.1 (1.7), respectively, and the mean decrease in score from baseline to six weeks in the duloxetine group was 1.06. We hypothesized similar results, namely that the mean NRSN before treatment would be 6.0 (1.7) and the mean decrease in score would be 1.0. We needed a target sample size of 23 participants to complete this protocol treatment in this study to obtain 80% power and a 2-sided α of 5% to detect the target change using the paired sample *t*-test. Assuming a dropout rate of 20%, the sample size in our study was set at 30 patients.

The secondary endpoints and subgroup analyses were analyzed using the dependent *t*-test or Wilcoxon's signed-rank test according to the statistical distribution for continuous variables, and Fisher's exact test for categorical variables. All statistical analyses were performed with SAS software version 9.4.

## Results

### Patient characteristics and treatment

We enrolled a total of 34 patients for this study between September 2014 and December 2015. Patient characteristics at baseline are summarized in Table 1. Two patients (6%) were concomitantly receiving adjuvant analgesic agents for CIPN, including pregabalin, goshajinkigan, and duloxetine, and four patients (12%) were concomitantly receiving controlled-release oxycodone for cancer-related pain. None of the patients was receiving both the adjuvant analgesics and oxycodone.

**Table 1.** Patient Baseline Characteristics (*n* = 34)

Age (range), years	67 (46–74)
Gender: male/female	22/12
Primary disease, *n* (%)	
Colorectal	19 (55.9)
Pancreas	12 (35.3)
Stomach	3 (8.8)
Causative agent, *n* (%)	
Oxaliplatin	20 (58.8)
Nab-paclitaxel	10 (29.4)
Oxaliplatin and nab-paclitaxel	2 (5.9)
Paclitaxel	2 (5.9)
Concomitant drugs for cancer-related pain, *n* (%)	
NSAIDs	5 (14.7)
Other opioids	4 (11.8)
Adjuvant analgesics	2 (5.9)
Neurotoxic chemotherapy, *n* (%)	
Concurrent use	25 (73.5)
Previous use	9 (26.5)
Numerical rating scale of neuropathic pain	
Mean ± SD (range)	5.53 ± 1.88 (3–8)
Peripheral sensory neuropathy (CTCAE v4.0)	
Grade 2/3/4	19/15/0

CTCAE, common terminology criteria for adverse events; NSAIDs, nonsteroidal anti-inflammatory drugs; SD, standard deviation.

### Efficacy

**FIG. 1.**
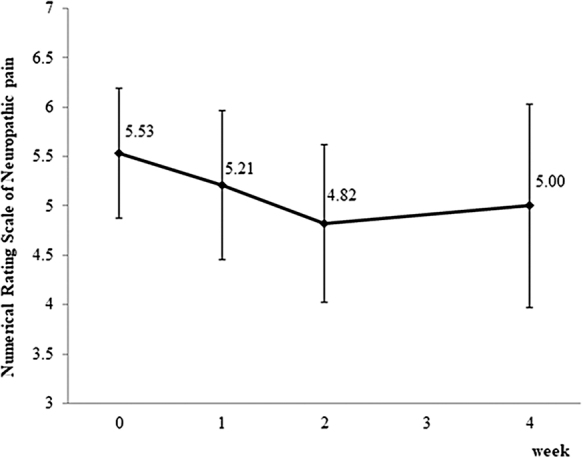
Change in mean NRSN. NRSN during the treatment period tended to be lower than the score at baseline. The mean change from baseline to day 28 was 0.53 (95% CI −0.21 to 1.43; *p* = 0.139; by paired *t*-test). Therefore, the primary endpoint was not met in this study. Error bars represent 95% CIs. CI, confidence interval; NRSN, numerical rating scale of neuropathic pain.

**FIG. 2.**
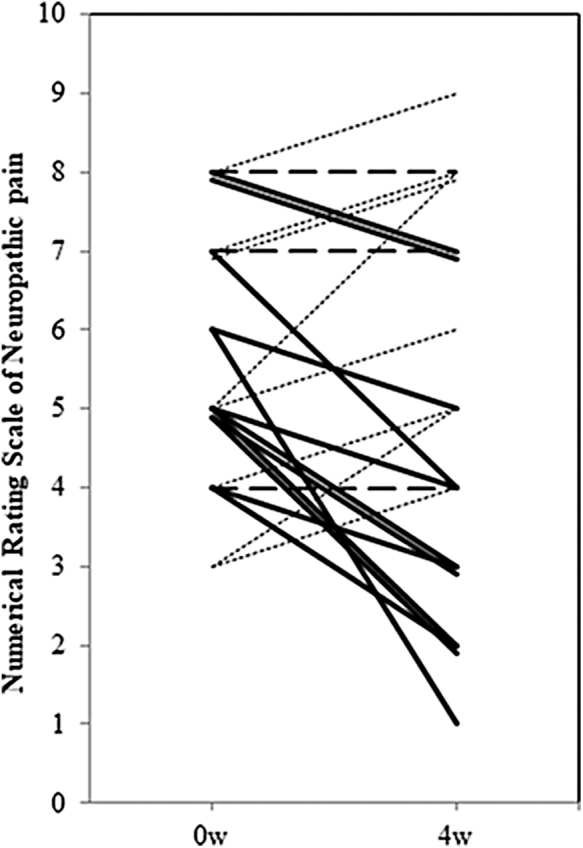
NRSN at baseline and after treatment. Thirteen of 23 patients who completed the protocol treatment showed an improvement in NRSN by ≥1 point from baseline to the end of the 28-day treatment period (solid lines). In three patients, the neuropathic pain score showed no change (broken lines). In seven patients, the neuropathic pain score increased compared with the baseline score (dotted lines).

All 34 participants received the intervention and were evaluated for efficacy and safety. The mean NRSN (SD) decreased from 5.53 (1.88) at baseline to 5.00 (2.37) on day 28 (Fig. 1). The mean decrement was 0.53, which was not statistically significant (95% confidence interval −0.21 to 1.43; *p* = 0.139). However, 13 of the 23 patients (57%), who completed the experimental treatment, showed an improvement in neuropathic pain score by at least 1 point (Fig. 2). There was no significant difference in efficacy between patients who were currently receiving the neurotoxic chemotherapies and those who had previously received them. Other analyses in subgroups classified according to the primary location of the cancer, the causative neurotoxic agents (L-OHP or taxanes), and the concomitant use of other opioids or adjuvant analgesics revealed no statistically significant results.

### Adverse events

Eight events in seven patients (21%) were reported, namely nausea in three (8.8%), somnolence in three (8.8%), and malaise in two (5.9%). One patient had both somnolence plus malaise. In each AE, the clinical grade was 1 or 2 according to CTCAE v4.0, and there were no cases of any severe AE. However, all seven patients who encountered AEs discontinued the treatment. The median time of onset of AEs was day 7 (2–26) of treatment, and five patients discontinued the protocol treatment within seven days.

### Genetic polymorphism of CYP2D6

Consent for biomarker research was obtained from 25 participants, and SNPs were examined. In this biomarker analysis cohort, 17 patients completed the study treatment. The mean NRSN (SD) in the cohort was 5.76 (1.52) at baseline and 5.00 (2.24) on day 28.

Frequencies of the *CYP2D6* SNPs are given in Table 2, and the distribution of the genotype and phenotype (enzyme activity) is summarized in Table 3. The allele frequencies were similar to those in a previous Japanese report.^9^ No UMs or PMs were identified in this study, because there were no patients with an active allele (duplication of *1, *2) or homozygous nonfunctioning alleles (e.g., *5/*5). Seven (28%) of the 25 patients had two active alleles (*1/*1, *1/*2, *2/*2), and 12 patients (48%) had just one active allele (*1/*5, *1/*10, *2/*10, *2/*41) that resulted in the EM phenotype. Six patients (24%) had two alleles with a reduction in enzyme function (*5/*10, *10/*10, *10/*41), which resulted in the IM phenotype. Among these six IM patients, two patients completed the study treatment, both of whom showed improved scores on neuropathic pain (−1.0 and −2.0, respectively); two patients discontinued the protocol treatment due to the toxicity of TACT; and the remaining two patients discontinued the treatment due to transfer to other hospitals. Our findings suggested that tramadol/acetaminophen may be more effective in patients with the IM phenotype although at the cost of increased toxicity.

**Table 2.** Frequencies of *CYP2D6* SNPs in This Study

	Enzyme function	Frequency (%)
CYP2D6^*^1	Normal	30
CYP2D6^*^2	Normal	22
CYP2D6^*^5	None (*CYP2D6* deleted)	8
CYP2D6^*^10	Reduced function	36
CYP2D6^*^41	Reduced function	4

Duplication of CYP2D6^*^1 or ^*^2 was not observed.

**Table 3.** Effect of *CYP2D6* Polymorphisms on Neuropathic Pain and Toxicity

Genotype (n = 25)	Enzyme activity	n (%)	Completion of treatment (%)	Mean change in NRSN	Responders^a^ (%)	Discontinued because of toxicity (%)
^*^1/^*^1	EM	7 (28)	6 (86)	−0.67	8 (53)	3 (16)
^*^1/^*^2						
^*^2/^*^2						
^*^1/^*^5		12 (48)	9 (75)			
^*^1/^*^10						
^*^2/^*^10						
^*^2/^*^41						
^*^5/^*^10	IM	6 (24)	2 (33)	−1.50	2 (100)	2 (33)
^*^10/^*^10						
^*^10/^*^41						

^a^ Patients who showed an improvement in NRSN by at least 1 point.

EM, extensive metabolizer; IM, intermediate metabolizer; NRSN, numerical rating scale of neuropathic pain.

## Discussion

CIPN is one of the most distressing adverse effects associated with cancer chemotherapy. The American Society of Clinical Oncology (ASCO) guidelines for CIPN recommend only duloxetine (one of SNRIs) in the treatment setting (moderately strong evidence).^11^ One retrospective study suggested that oxycodone (opioid analgesic) might be effective against CIPN.^12^ Tramadol has a dual mechanism of action (opioid and SNRI action). Therefore, we hypothesized that TACT, which included acetaminophen in addition, might be effective, and conducted this phase II study.

Although the primary endpoint of improvement in mean NRSN was not met in this study, some clinical benefit was nevertheless obtained in more than half of the patients. The NRSN improved in 13 (57%) of the 23 patients who completed the protocol treatment. As a reference data, the duloxetine study reported that neuropathic pain decreased in 59% of the patients treated with duloxetine and 38% of the patients treated with placebo.^5^ Six of these patients were willing to continue the treatment after the study period due to its efficacy, and two patients resumed intake of TACT later because they felt that the neuropathic pain had gotten worse after terminating the protocol treatment on completion of the clinical trial period. Changes of CIPN were not evaluated by the CTCAE in this study.

SNPs of *CYP2D6*, which is recognized as biomarkers for the metabolism of tramadol, were measured in 25 patients (74%). CYP2D6 metabolizes tramadol to M1, which has 175-fold higher affinity for the μ-opioid receptor than tramadol.^13^ This finding suggests that the analgesic effect of tramadol is lower in PM cases due to poor enzyme activity, whereas both its pain-relief effect and opioid-related toxicity are higher in UM than in the other metabolizer phenotypes.^14^ To our knowledge, the findings of tramadol for CIPN associated with *CYP2D6* variations have not been reported. Although we saw neither PM nor UM cases in this study, 6 patients were categorized into the IM group (reduced metabolic function) and 19 patients were categorized into the EM group (normal metabolic activity). Because only two patients in the IM group completed this protocol treatment, this study could not show a significant difference in improvement of neuropathic pain scores between the IM and EM groups. However, there was a tendency for tramadol to show more efficacy in the IM patients than in the EM patients. It has been reported that tramadol compound has a stronger serotonin-reuptake inhibitor effect than M1.^4^ Because this SNRI effect impacts its efficacy against CIPN, the potential of *CYP2D6* polymorphism as a biomarker requires further study.

Although AEs were observed in seven patients (21%), no case had grade 3 or higher severity. All these patients with AEs discontinued the protocol treatment. It was assumed that all AEs in this study had been caused by tramadol, not by acetaminophen. Nausea is one of the most frequently encountered toxicities of tramadol, although in this study the incidence (8.8%) was lower than expected. Concurrent use of a 5-HT_3_ receptor blocker, which is frequently used as a prophylactic antiemetic in patients receiving cancer chemotherapy, might be effective in reducing tramadol-induced nausea, as the nausea is caused by the increase in the serotonin levels in synaptic clefts resulting from the SNRI action of tramadol. In this study, if a patient discontinued taking tramadol/acetaminophen once because of an AE(s), he/she could restart the protocol treatment after the AE(s) had improved. However, none of the patients made an attempt to resume treatment, and the discontinuation rate of the study treatment was higher than expected.

This study had several limitations. First, the number of evaluable patients who completed the protocol treatment was small. Some patients experienced benefit from TACT, although promising factors could not be identified in the subgroup analyses because the number of patients in each subgroup was too small. Second, this study was not a randomized or blinded trial.

## Conclusions

Although this study did not meet its primary endpoint, the severity of neuropathic pain was reduced in more than half of the patients. In current practice, TACT could be considered as a valid treatment option in patients with CIPN in whom duloxetine proves ineffective.

## Abbreviations Used

ADL: activities of daily living
AEs: adverse events
CIPN: chemotherapy-induced peripheral neuropathy
CI: confidence interval
CTCAE: Common Terminology Criteria for Adverse Events
CYP2D6: cytochrome P450 2D6
EM: extensive metabolizer
IM: intermediate metabolizer
nab-PTX: nanoparticle albumin-bound paclitaxel
NSAID: nonsteroidal anti-inflammatory drug
NRSN: numerical rating scale of neuropathic pain
L-OHP: oxaliplatin
PTX: paclitaxel
PM: poor metabolizer
SNRI: serotonin-noradrenaline reuptake inhibitor
SNPs: single nucleotide polymorphisms
SD: standard deviation
TACT: tramadol/acetaminophen combination tablet
UM: ultrarapid metabolizer
